# Roles of Glutathione in Mediating Abscisic Acid Signaling and Its Regulation of Seed Dormancy and Drought Tolerance

**DOI:** 10.3390/genes12101620

**Published:** 2021-10-14

**Authors:** Murali Krishna Koramutla, Manisha Negi, Belay T. Ayele

**Affiliations:** Department of Plant Science, 222 Agriculture Building, University of Manitoba, Winnipeg, MB R3T 2N2, Canada; murali.koramutla@umanitoba.ca (M.K.K.); Manisha.Negi@umanitoba.ca (M.N.)

**Keywords:** abscisic acid, glutathione, dormancy, germination, stomatal closure, drought

## Abstract

Plant growth and development and interactions with the environment are regulated by phytohormones and other signaling molecules. During their evolution, plants have developed strategies for efficient signal perception and for the activation of signal transduction cascades to maintain proper growth and development, in particular under adverse environmental conditions. Abscisic acid (ABA) is one of the phytohormones known to regulate plant developmental events and tolerance to environmental stresses. The role of ABA is mediated by both its accumulated level, which is regulated by its biosynthesis and catabolism, and signaling, all of which are influenced by complex regulatory mechanisms. Under stress conditions, plants employ enzymatic and non-enzymatic antioxidant strategies to scavenge excess reactive oxygen species (ROS) and mitigate the negative effects of oxidative stress. Glutathione (GSH) is one of the main antioxidant molecules playing a critical role in plant survival under stress conditions through the detoxification of excess ROS, maintaining cellular redox homeostasis and regulating protein functions. GSH has recently emerged as an important signaling molecule regulating ABA signal transduction and associated developmental events, and response to stressors. This review highlights the current knowledge on the interplay between ABA and GSH in regulating seed dormancy, germination, stomatal closure and tolerance to drought.

## 1. Introduction

Plants are sessile in nature and are constantly exposed to a variety of environmental conditions that negatively affect their growth, development and productivity. However, they have evolved efficient signal perception and transduction mechanisms to combat these factors and sustain their growth and development. Previous studies have shown the involvement of phytohormones and other signal molecules in regulating plant developmental processes and their response to stress factors [1,2]. Abscisic acid (ABA) is one of the classical phytohormones considered to be a major player in this regard [3]. The response of plants to abiotic stressors usually involves activation of the ABA signaling cascade to mediate the transcription of downstream genes that regulate stress tolerance [4,5]. Genetic studies have revealed that the loss of function of genes involved in ABA metabolism or signaling affects plant developmental processes and leads to a decrease in stress tolerance [6,7]. The role of ABA in regulating plant development and stress response also involves its crosstalk with other phytohormones and signaling molecules such as glutathione (GSH) [8,9]. 

Glutathione is a water-soluble low molecular weight non-protein tripeptide (γ-glutamyl-cysteinyl-glycine) that serves as the most abundant thiol source in plant cells [10]. It is involved in several plant growth and developmental processes including regulation of cellular redox homeostasis and gene expression, and plant response to biotic and abiotic stress factors [11,12,13]. One of the major functions of GSH is detoxification of excess reactive oxygen species (ROS) produced by cellular processes under stress conditions [14,15]. As an antioxidant, GSH is a key component of the major H_2_O_2_ scavenging ascorbate-glutathione (AsA-GSH) metabolic pathway, acting as reducing agent to regenerate reduced ascorbate from its oxidized form via a reaction catalyzed by dehydroascorbate reductase (DHAR) [16]. Furthermore, GSH is involved in ROS removal by acting as a substrate in metabolic reactions catalyzed by glutathione-S-transferases (GST) and glutathione peroxidases (GPX) [15]. Under normal/non-stress conditions, plants maintain a relatively high cellular level ratio of reduced GSH to its oxidized form GSSG (GSH/GSSG) at a cellular level, however, under stress conditions, GSH is actively involved in antioxidant defense responses and it is converted to its oxidized form, GSSG. Previous studies have shown that intracellular GSH/GSSG ratio triggers signal transductions mediating plant response to stress factors and acts as a marker of intracellular redox homeostasis [17,18]. In support of this, several studies have demonstrated the role of GSH in enhancing plant tolerance to a variety of abiotic stressors including drought, cold, salinity, high temperature and metal toxicity [19,20,21]. Plant hormones such as ABA are implicated in modulating the intracellular GSH/GSSG ratios under abiotic stress conditions [22,23]. For example, exogenous ABA has been shown to increase the ratio of GSH/GSSG levels in tomato plants [24]. Furthermore, ABA has been shown to lead to differential alteration of the GSH/GSSG ratios in two maize lines with varying degree of tolerance to osmotic and drought stress [25]. GSH is also involved in post-translational modification of proteins through *S*-glutathionylation and *S*-nitrosoglutathione (GSNO)-mediated *S*-nitrosylation, and these protein modifications modulate signaling events and thereby influence plant developmental processes and response to stress factors [23].

The roles of ABA or GSH in either plant development or stress response have been reviewed extensively; however, literature on the role of coordinated interplay between ABA and GSH in regulating both plant developmental processes and plant-environment interactions are scarce. This review highlights the current knowledge on the role of crosstalk between GSH and ABA signaling in regulating plant development and stress response with emphasis on seed dormancy, germination, stomatal closure and drought tolerance. 

## 2. Abscisic Acid Metabolism and Signaling

### 2.1. Abscisic Acid Metabolism

ABA in plants is synthesised from zeaxanthin through several enzymatic reactions [26]. Zeaxanthin (C_40_) is first converted to xanthoxin (C_15_) by the actions of zeaxanthin epoxidase (ZEP) and 9-*cis*-epoxycarotenoid dioxygenase (NCED). The NCED-mediated reaction, which involves a non-reversible oxidative cleavage of 9-*cis*-violaxanthin and/or 9-*cis*-neoxanthin to xanthoxin, is considered the rate-limiting step in ABA biosynthesis. Xanthoxin dehydrogenase converts xanthoxin to abscisic aldehyde, which is further oxidized to ABA by the action of abscisic aldehyde oxidase (AAO).

ABA catabolism also plays an important role in regulating ABA levels in plant tissues. ABA catabolism involves two types of reactions, namely hydroxylation and conjugation [26]. The main hydroxylation reaction of ABA catabolism involves the conversion of ABA to phaseic acid through hydroxylation of the C-8′ methyl group of the ABA ring structure by the action of ABA 8′-hydroxylase, which is encoded by *CYP707As* genes. ABA catabolism by conjugation involves the formation of ABA-glucosyl ester (ABA-GE) by the action of UDP-glucosyltransferase. Upon cellular demands, inactive ABA-GEs can be rapidly converted to active ABA by ABA-GE hydrolyzing enzymes, beta-glycosidases [27].

### 2.2. ABA Signaling

ABA signaling in plants involves three core components, namely, pyrabactin resistance (PYR)/pyrabactin resistance-like (PYL)/regulatory component of ABA receptors (RCAR) (PYR/PYL/RCAR), type 2C protein phosphatases (PP2Cs), which act as negative regulators of ABA signaling, and sucrose non-fermenting-1-(SNF1)-related protein kinase 2 (SnRK2) that acts as a positive regulator of ABA signaling [28,29]. In the absence of ABA, PP2Cs bind to and inactivates SnRK2, thereby inhibiting ABA signaling; however, in the presence of ABA, the PYR/PYL/RCAR forms a complex with PP2Cs, leading to inhibition of PP2Cs activity and thereby activating SnRK2 [30]. The activated SnRK2 phosphorylates thereby activates the downstream transcription factors including the ABA insensitive 3 (*ABI3*), *ABI4* and *ABI5* that belong to the B3, AP2 and basic leucine zipper-(bZIP) domain transcription factor families, respectively, which regulate the expression of ABA responsive genes. 

## 3. Glutathione Biosynthesis and Metabolism

The synthesis of GSH from its constituent amino acids, which include glutamate, L-cysteine and glycine, involves two ATP-dependant enzymatic reactions mediated by γ-glutamylcysteine synthetase (γ-ECS or GSH1) and GSH synthetase (GS or GSH2) [31]. The γ-ECS enzyme catalyzes the first and rate-limiting step to produce γ-glutamylcysteine (γ-EC) from the L-glutamate and L-cysteine amino acids. In the second step, GSH synthetase catalyzes the addition of glycine to γ-glutamylcysteine (γ-EC) to produce GSH. The reaction catalyzed by γ-ECS/GSH1 is considered as the rate-limiting step of GSH synthesis, and the activity of this enzyme is regulated by cellular levels of cysteine and glutamic acid and feedback inhibition by γ-EC and GSH [32]. 

**Figure 1:**
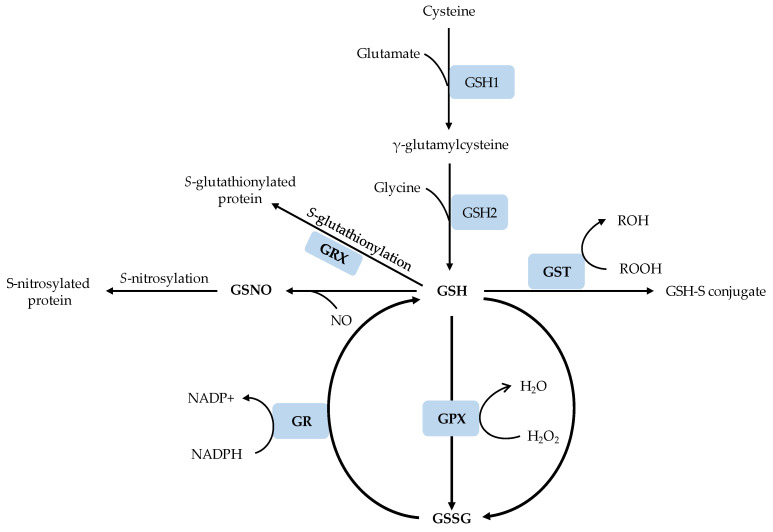
A schematic representation of GSH biosynthesis and metabolism. GSH1, γ-glutamylcysteine synthetase; GSH2, GSH synthetase; GSH, reduced glutathione; GSSG; oxidized glutathione; GST, glutathione-S-transferases; GPX, glutathione peroxidase; GRX, glutaredoxin; GR, glutathione reductase; GSNO, *S*-nitrosoglutathione.

GSH1 is localized only in the plastids while GSH2 is localized in the plastids and the cytosol, and both GSH1 and GSH2 are encoded by a single gene [33] (Figure 1). Consistent with the localization of GSH1, the first step of GSH synthesis occurs in the plastids; however, since the most abundant transcript of the multiple GSH2 transcript populations encodes a cytosolic GSH2, the second step is reported to occur most likely in the cytosol [15]. After its synthesis in the cytosol, GSH can be transported to other cellular compartments, predominantly in its reduced form or conjugated forms [34]. The reduced GSH can rapidly be converted to its oxidized form, GSSG, in various biochemical reactions, and the cellular homeostasis of GSH/GSSG ratio is maintained by the actions of glutathione reductase (GR) and GPX [35]. Deficiency in the activity of either GSH1 or GSH2 impairs GSH production and thereby negatively affects plant growth and development. For example, the Arabidopsis GSH1 knockout mutants, *gsh1* and *rml1* (root meristem less 1), are characterized by low GSH content, poor postembryonic root development and embryo death [36,37,38]. Several factors including the concentrations of cysteine and glycine, availability of ATP, photosynthetically active photon flux and enzymes that consume GSH also regulate the biosynthesis of GSH [15,39]. GSH, once produced, can also be subjected to conjugation with toxic xenobiotic substances by the action of GST [40] or can serve as a substrate for S-glutathionylation of proteins in the presence of small redox enzyme glutaredoxins (GRX), which also uses GSH as a cofactor (Figure 1). GSH can also react with NO free radical to produce GSNO, which nitrosylates target proteins.

## 4. Glutathione Modulates ABA Signaling in the Regulation of Seed Dormancy and Germination

Seed dormancy and germination are important developmental processes that are regulated by several signaling pathways [41]. Plant hormones, mainly ABA and gibberellin (GA), play central roles in regulating the induction, maintenance and release of seed dormancy and seed germination [29]. Other signal compounds including ROS, such as H_2_O_2_, are also implicated in the control of these developmental events via regulating ABA-GA balance [42,43]. However, excess production of ROS can lead to oxidative stress that negatively affects plant growth and developmental processes. Thus, activation of antioxidant systems to scavenge the excessive ROS molecules is crucial to mitigate the negative effects. 

Glutathione is one of the most important components of antioxidant systems that scavenge excessive ROS molecules. Owing to this role, GSH could play a role in the control of seed dormancy and germination. It has been shown previously that GSH breaks seed dormancy in barley, and dormancy breakage by H_2_O_2_ was shown to be associated with enhanced GSH level [44]. Similarly, treatment of recalcitrant seeds of silver maple with GSH has been shown to decrease the rate of dehydration and stimulate germination [45]. This was associated with a higher level of H_2_O_2_, which is proposed to have a signaling function to help the seeds cope with water stress associated with the dehydration process instead of acting as an indicator of severe oxidative stress and enhanced AsA-GSH cycling [45]. Previous studies have shown that enzymes which regulate the GSH pool have a significant effect on ABA signaling and the regulation of seed germination and dormancy. For example, genes encoding GPX and GST enzymes, which modulate cellular GSH levels, have been shown to play roles in regulating ABA signaling in arresting seed germination [46,47,48,49]. However, an increase in the levels of GSH-containing glutathionylated proteins/compounds such as GRX and GSNO have been shown to suppress ABA signaling during seed germination [50,51].

### 4.1. Glutathione Peroxidase and Glutathione S-Transferase as Regulators of GSH Pool and ABA Signaling in the Control of Seed Dormancy and Germination

Glutathione peroxidase is an antioxidant enzyme that protects plants from oxidative stress via reducing lipid peroxides and free H_2_O_2_ to their corresponding alcohols and water by utilizing GSH [52]. GPX also plays an important role in regulating ABA signaling [46,49]. It has been shown previously that the Arabidopsis *GPX* loss of function mutants *gpx3-1* and *gpx3-2*, which lack GPX activity, are insensitive to ABA during seed germination. This role of *GPX* in regulating ABA response is mediated by its modulation of the activity of ABI proteins, mainly ABI2 that acts as a negative regulator of ABA signaling [46]. Furthermore, seeds from *GPX3* silenced rice plants (*gpx3i*) are insensitive to ABA and showed germination in the presence of ABA, while germination of seeds from the corresponding wild-type/control plants was completely inhibited by ABA [49]. The *gpx3i* mutant plants are also characterized by the prevalence of enhanced glutathionylation, repressions of proteins involved in epigenetic regulation and ubiquitination, and upregulation of the PP2C protein [49]. In contrast, ectopic expression of the putative wheat *GPX* genes, designated as *W69* and *W102*, in Arabidopsis has been reported to exhibit reduced seed sensitivity to ABA and enhanced germination under high salt stress [53]. Possible reasons for this contradictory result include differences in the concentration of exogenous ABA, plant growth conditions, type of *GPX* gene homologs and the plant species considered in the respective studies. These results therefore highlight the multifunctionality of GPX isoenzymes that are known to have distinct subcellular locations; their genes exhibit distinct expression patterns in response to different environmental factors or in different plant species [52]. However, alterations in the expression levels of the ABA signaling genes *ABI1* and *ABI2* and the ROS biosynthesis gene *RbohD* in *GPX* overexpressing transgenic plants, and induction of PP2C protein in *GPX3* silenced plants along with the observation of physical interaction between GPX and ABI proteins, highlight the role of GPX in modulating ABA signaling and thereby seed dormancy and germination. 

Glutathione S-transferase is a ubiquitous protein that decreases the GSH pool via catalysing the conjugation of GSH to various xenobiotics to detoxify such compounds, which accumulate as a result of oxidative stress, and thereby maintain cellular redox homeostasis [40]. Therefore, GSTs affect a range of redox-dependent cellular processes that involve hormone and stress responses including ROS-mediated ABA metabolism and signaling. Consistently, the *gstu7* and *gstu17* mutants of Arabidopsis have been reported to exhibit increased GSH and ABA levels and decreased H_2_O_2_ levels, and the seeds of these mutants are found to be less sensitive to ABA during germination [47,48]. Furthermore, the *gstu7* mutant shows reduction in the expression levels of genes encoding proteins that act as positive regulators of ABA signaling including *SnRK*, *ABI3* and *ABI5* [48]. In contrast, overexpression of *GSTU19* has been shown to lead to induction of germination under drought conditions and this effect is associated with increased levels of proline and activities of antioxidant enzymes [54]. Similarly, ectopic expression of the rice *GSTU4* gene in Arabidopsis has been reported to lead to enhanced seed germination under salinity and oxidative stress conditions [55]. The same authors also showed that the transgenic Arabidopsis plants expressing rice *GSTU4* exhibit reduced ABA sensitivity and ROS levels. Seeds of Arabidopsis plants expressing the *GST* gene of *Tamarix hispida* (*GSTZ1*) are also shown to be less sensitive to ABA during germination [56]. These results imply the importance of GSH-ROS homeostasis in ABA-mediated regulation of seed dormancy and germination. 

### 4.2. Glutathione-Mediated Post-Translational Control of ABA Signaling, and Seed Dormancy and Germination

Glutaredoxins are thiol-disulfide oxidoreductases (thioltransferases) that use the reducing power of GSH to catalyse the reversible reduction of disulfide bonds of substrate proteins, leading to their post-translational modification. They also function in scavenging cellular ROS and regulating redox homeostasis [57,58]. Genetic studies that involved overexpression or silencing of the *GRX* genes have demonstrated their importance in the regulation of plant oxidative stress responses [51,59]. Glutaredoxins have been implicated as negative regulators of ABA signaling during seed germination/preharvest sprouting [51,59,60]. The rice *PHS9* gene encodes a unique CC-type *GRX* that interacts with putative GTPase activating protein (GAP), which is an interacting partner of the ABA receptor RCAR1, and overexpression of either *PHS9* or *GAP* has been shown to lead to reduced ABA sensitivity during seed germination [51]. Similarly, seeds of Arabidopsis plants expressing the cassava gene encoding the CC-type GRX-C15 (designated as *GRXC15*) have been shown to exhibit enhanced germination in the presence of ABA, indicating decreased seed sensitivity to ABA [59]. Consistently, silencing of the *GRXS17* gene in rice leads to seed hypersensitivity to ABA and thereby the inhibition of germination; however, the germination inhibitory effect of ABA can be reversed by GSH [60]. Since glutaredoxins are known to be important components in maintaining redox-dependent signaling [61], it is likely that silencing *GRXS17* leads to increase in the accumulation of H_2_O_2_, which is known to positively regulate ABA signaling and thereby inhibit seed germination. In agreement with this, accumulation of H_2_O_2_ was prevalent in the root tips of *GRXS17*-silenced rice seedlings [60].

**Figure 2:**
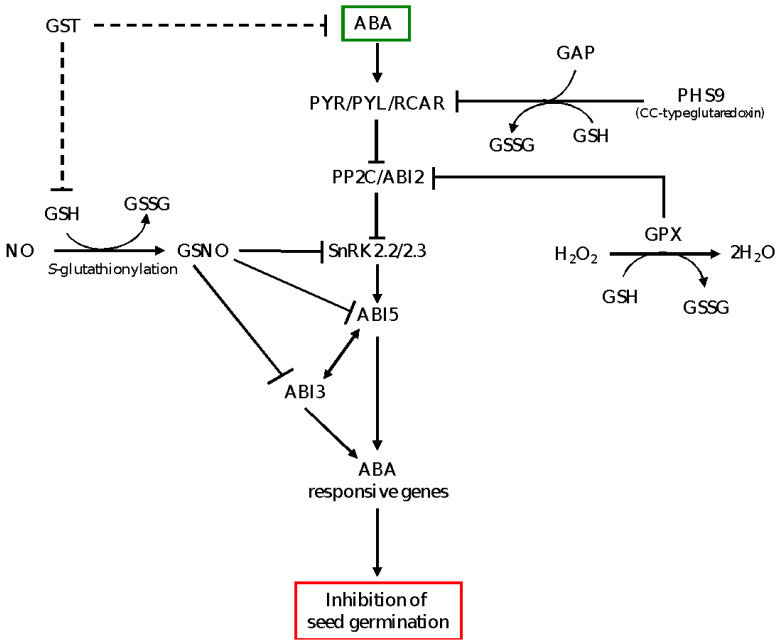
A model depicting GSH-mediated ABA signaling in the control of seed dormancy and germination. GSH in the presence of NO forms GSNO, which inhibits SnRK2.2/2.3 and ABI5 proteins of ABA signaling through *S*-nitrosylation, leading to repression of ABA signaling and promotion of seed germination. The CC-type glutaredoxin (PHS9) interacts with putative GTPase activating protein (GAP), which acts as an interacting partner of RCAR and inhibits PYR/PYL/RCAR, thereby repressing ABA signaling and enhancing seed germination. The GPX, which acts as antioxidant enzyme in maintenance of H_2_O_2_ homeostasis, physically interacts with and inhibits ABI2, a negative regulator of ABA signaling, leading to activation of ABA signaling and inhibition of seed germination. GST is proposed to reduce the levels of ABA and GSH (broken lines); GSH suppresses the expression of ABA signaling genes and therefore induces seed germination. PYR/PYL/RCAR, pyrabactin resistance/pyrabactin resistance-like/regulatory component of ABA receptors; PP2C, type 2C protein phosphatase; SnRK, sucrose non-fermenting-1-related protein kinase; ABI2/3/5, ABA insensitive 2/3/5; NO, nitric oxide; GSNO, S-nitrosoglutathione; GST, glutathione S transferase; GPX, glutathione peroxidase; GSH, reduced glutathione; GSSG, oxidized glutathione.

Previous studies have shown that the role of NO in promoting seed germination is associated with negative regulation of ABA signaling via inhibition of SnRK and S-nitrosylation-mediated degradation of ABI5 [50,62]. Furthermore, NO has been shown to break seed dormancy through inducing the expression levels of ABA catabolic gene *CYP707A2* and thereby reducing ABA level [63]. GSH plays a critical role in the regulation of NO signaling as it reacts with NO and forms GSNO [64], an important mobile cellular NO that triggers NO signal transduction [65]. Consistently, dormancy release in response to the cold stratification of apple seeds has been reported to be associated with enhanced levels of GSNO and GSH [66]. These reports highlight the significance of GSH in mediating NO and ABA cross-signaling in the regulation of seed dormancy and germination, although further studies are required to elucidate such functionality of GSH and the associated underlying mechanisms. The role of GSH in mediating ABA signaling in the control of seed dormancy and germination is depicted by a model shown in Figure 2. 

## 5. Glutathione and ABA-Induced Stomatal Closure

### 5.1. Modulation of Glutathione Level and Its Role in ABA-Mediated Stomatal Closure

Stomata, small pores on the epidermis of the leaf, regulate transpiration, photosynthetic gas exchange and microbial entry. Stomatal closure represents the initial defense response of plants to biotic and abiotic stresses, for example, to restrict microbial entry or water loss. Stomatal guard cells are able to sense and respond to external stimuli and thereby quickly regulate stomatal aperture [67]. Several plant hormones are known to influence stomatal closure in order to reduce transpirational water loss under water deficit/drought conditions. However, the plant hormone ABA acts as a major regulator of this event by involving other signaling components such as ROS, NO, reactive carbonyl species (RCS) and Ca^2+^ in the guard cells [67,68]. 

Previous studies have also implicated GSH in ABA-mediated stomatal closure. It has been reported that the Arabidopsis *chlorinal-1* (*ch1-1*) mutant, which is deficient in light-harvesting complex proteins in photosystem II, exhibits reduced levels of GSH in the guard cells [69,70]. While stomatal closure in the *ch1-1* mutant is enhanced by exogenous ABA, treatment with exogenous GSH leads to inhibition of stomatal closure even after ABA treatment [69]. These results suggest that a decrease in GSH level in the guard cells promotes ABA-mediated stomatal closure. However, GSH appears not to affect ABA-induced ROS production since treatment of the *ch1-1* mutant with exogenous GSH donor does not have significant effect on ABA-induced production of ROS. Similarly, *cad2-1* mutant of Arabidopsis, which is defective in γ-ECS activity, exhibits a reduced level of GSH in the guard cells and thereby induction of ABA-mediated stomatal closure [8]. However, a recent report has shown that stomatal closure in the *cad2-1* mutant is not associated with reduction in GSH level, but rather with accumulation of cysteine, a precursor of GSH [71]. Cysteine is the product of the sulfate assimilatory pathway, and previous reports have provided insights into the role of sulfur assimilation into cysteine in regulating ABA level and signaling, and thereby stomatal closure [71,72]. Previous studies have implicated that cysteine induces transcriptional activation of NCED3 and also serves as a source of sulfur for molybdenum cofactor (MoCo) sulfurylase (ABA3)-mediated production of sulfurylated form of (MoCo), which is crucial for the activation of the ABA biosynthesis enzyme AAO3, leading to enhanced ABA biosynthesis [71,73]. Furthermore, ABI1 and SnRK have been implicated as critical ABA signaling components in mediating cysteine-induced stomatal closure [72]. Therefore, a decrease in GSH synthesis in the guard cells leads to cysteine accumulation, which in turn activates ABA biosynthesis and signaling, and therefore stomatal closure. 

Other evidence that supports the induction of stomatal closure in response to a decrease in GSH level comes from studies that involve the use of chemicals that deplete the GSH level such as iodomethane, p-nitrobenzyl chloride and ethacrynic acid. Exogenous applications of such chemicals to excised leaves of Arabidopsis have been shown to reduce intracellular GSH levels in the guard cells [74]. This is shown to be associated with the prevalence of enhanced ABA-induced stomatal closure without any change in the cytosolic alkalization, cytosolic Ca^2+^ oscillation and ROS production in the guard cells [74]. It is therefore likely that the role of GSH in the guard cells in regulating stomatal closure is not associated with the scavenging of ABA-induced ROS accumulation but is via its effect on ABA signaling components downstream of ROS generation. Overall, these reports indicate the role of modulation of GSH level in the regulation of ABA-mediated stomatal closure and therefore tolerance to drought.

### 5.2. Glutathione Peroxidase as a Regulator of GSH Pool and ABA-Induced Stomatal Closure

It has been reported that H_2_O_2_ plays a key role in ABA-induced stomatal closure through affecting the Ca^2+^ channels in the guard cells [75]. ABA signaling involves the binding of ABA to the PYR/PYL/RCAR receptor, which in turn interacts with PP2Cs that act as negative regulators of ABA signaling and thereby regulate the downstream components [76]. Mutation in *ABI1* disrupts ABA signaling upstream of H_2_O_2_ synthesis, whereas mutation in *ABI2* impairs signaling downstream of H_2_O_2_ production in the guard cells [77]. Previous study has shown that ABA-induced stomatal closure is regulated by GPX, an antioxidant enzyme that catalyzes the reduction of H_2_O_2_ by using GSH as a substrate. GPX3, which functions as redox transducer in H_2_O_2_ signal transduction, interacts with ABI2 and thereby directly influences guard cell plasma membrane Ca^2+^ channels in regulating ABA-induced stomatal closure [46]. Consistently, the *gpx3* mutant of Arabidopsis is less sensitive to ABA- and H_2_O_2_-induced stomatal closure [46]. Similarly, silencing of *GPX3* in rice makes plants less sensitive to ABA-induced stomatal closure [49]. Proteomic studies have also revealed that silencing of *GPX3* induces S-glutathionylation and inhibits protein ubiquitination [49]. The involvement of protein ubiquitination in ABA signaling is well established, for example, ABA signaling is activated by the degradation of ABI1, a negative regulator of ABA signaling, through the UBC27-AIRP3 ubiquitination complex [78]. In addition, the protein components involved in the ubiquitination and proteasome complex are reported to be S-glutathionylated at cysteine residues under stress conditions [79,80]. Overall, these reports indicate the significance of GSH redox pool in the guard cells of the stomata to the control of ABA-induced stomatal closure via post-translational modifications of ABA signaling components.

## 6. Glutathione-Mediated ABA Signaling in Drought Tolerance 

ABA plays a critical role in regulating plant responses to various unfavorable environmental conditions including drought stress [81]. An increase in ABA level in response to abiotic stress factors such as drought has been reported in many plant species [82]. In agreement with this, exogenous ABA or genetic mutations that lead to an increase in ABA level and signaling have been shown to improve the performance of plants under drought conditions. For example, treatment of plants with exogenous ABA or its synthetic analogues enhances drought tolerance in several species including wheat [83,84,85], barley [86], rice [87], sugarcane [88] and tea [89]. Furthermore, overexpression of the ABA biosynthetic gene NCED in tomato [90], tobacco, [91] and Petunia [92], and the ABA signaling gene PYL in rice [93] and tomato [94] results in improved tolerance to drought.

Tolerance of plants to drought and other abiotic stress factors is also mediated by other mechanisms such as those involving antioxidant defence systems that mitigate drought-induced oxidative stress. Plants exposed to abiotic stress factors such as drought generate excessive ROS, and this ROS is subjected to detoxification either through the enzymatic or non-enzymatic antioxidant systems. With respect to the non-enzymatic antioxidant system, the AsA-GSH pathway plays a central role in ROS scavenging. Previous studies have revealed a close relationship between ABA and GSH in mediating plant response to drought stress; early accumulation of ABA stimulates ROS production, which in turn enhances the expression level of several genes involved in the AsA-GSH pathway and GSH content to counter stress-induced oxidative stress [84]. It has been shown previously that the levels of non-enzymatic antioxidants such as GSH and ascorbate increases in response to drought in many plant species [84,95]. Furthermore, exogenous application of ABA, for example in wheat, maize and soybean, leads to upregulation of genes involved in GSH synthesis and/or an increase in GSH level, resulting in an enhanced tolerance to drought [84,96,97]. Conversely, treatment with exogenous GSH or overexpression of the GSH biosynthesis gene *GSH1* in Arabidopsis improves drought tolerance through altering the expression patterns of ABA metabolic and signaling genes, leading to upregulation of the downstream ABA responsive genes [12,47]. Despite these reports, the mechanisms underlying the interaction between GSH and ABA in regulating plant response to drought remain to be elucidated.

### 6.1. Glutathione Peroxidase and Glutathione S-Transferases as Regulators of GSH Pool and Drought-Induced ABA Signaling

Previous studies have provided insight into the roles of different enzymes such as GPX and GST that regulate GSH homeostasis in the regulation of ABA signaling and drought tolerance [21,46]. Loss of function mutation in *GPX3* of Arabidopsis results in an increase in H_2_O_2_ levels, interruption of ABA-activated calcium channels and repression of ABA and stress responsive genes, leading to increased sensitivity to drought, while overexpression of *GPX3* enhances tolerance to drought stress [46]. Consistently, the same authors have shown that GPX interacts with ABI proteins, highlighting its importance in mediating ABA and drought signaling. In addition, ectopic expression of *GPX* of *Rhodiola crenulata* (*GPX5*) in *Salvia miltiorrhiza* plants resulted in increased drought tolerance through enhancing GSH content and expression of ABA-signaling genes [21]. These reports suggest dual roles of GPX in H_2_O_2_ homeostasis; in scavenging H_2_O_2_ and regulating the use of H_2_O_2_ as an oxidative signal transducer in the modulation of ABA and drought stress signaling. 

Drought stress has been shown to induce the expression level of genes encoding GST and activity of the corresponding enzyme in several plant species [98,99]. Furthermore, ectopic expression of the rice *GSTU4* and *GSTU30* genes in Arabidopsis confers tolerance to drought and oxidative stress. These effects are closely associated with lower accumulation of ROS, upregulation of ABA responsive genes, including *ABI3*, *ABI5*, *CHYR1* and *RAB18*, that are known to have roles in plant response to drought stress, and decreased sensitivity to exogenous ABA [55,99]. These results suggest the role of *GSTU* genes in mediating the ABA-dependent oxidative and drought stress tolerance. In contrast, the Arabidopsis *GSTU17* has been shown to function as a negative regulator of drought-mediated signal transduction pathways; the *gstu17* mutant exhibits increased GSH and ABA levels, reduced stomatal aperture and transpirational water loss rates, leading to enhanced tolerance to drought [47]. Given that GST, which is encoded by large gene families, has multiple functions [100], its role in ABA-mediated drought stress signaling might vary with plant species. 

### 6.2. Glutathione-Mediated Post-Translational Control of ABA Signaling in Drought Tolerance

*S*-glutathionylation is a reversible redox-sensitive post-translational modification that adds GSH to cysteine residues of proteins and thereby modulates their functions [101]. This protein modification mechanism occurs especially under increased production of ROS and/or GSSG. Among the stress-related plant proteins that undergo *S*-glutathionylation are annexins, multi-functional calcium-dependent membrane binding proteins that serve as important components in calcium signaling pathways [102]. Overexpression of *annexin* genes in different plant species has been shown to enhance drought tolerance, and this effect is closely associated with reduced accumulation of ROS, increased ABA content and sensitivity, and thereby enhanced stomatal closure and reduced transpirational water loss [103,104,105]. Studies in Arabidopsis have revealed that *S*-glutathionylation of the cysteine residues in the AnnAt1 protein occurs in response to ABA treatment, and glutathionylation modification of AnnAt1 reduces its calcium-binding activity [103], which might affect its ability to form Ca^2+^ channels in biomembranes and thereby ABA-dependent drought signaling. Given that GSH regulates calcium influx [106,107], it is likely that GSH is involved in regulating drought tolerance through *S*-glutathionylation of proteins that mediate ABA-dependent drought signaling. 

**Figure 3:**
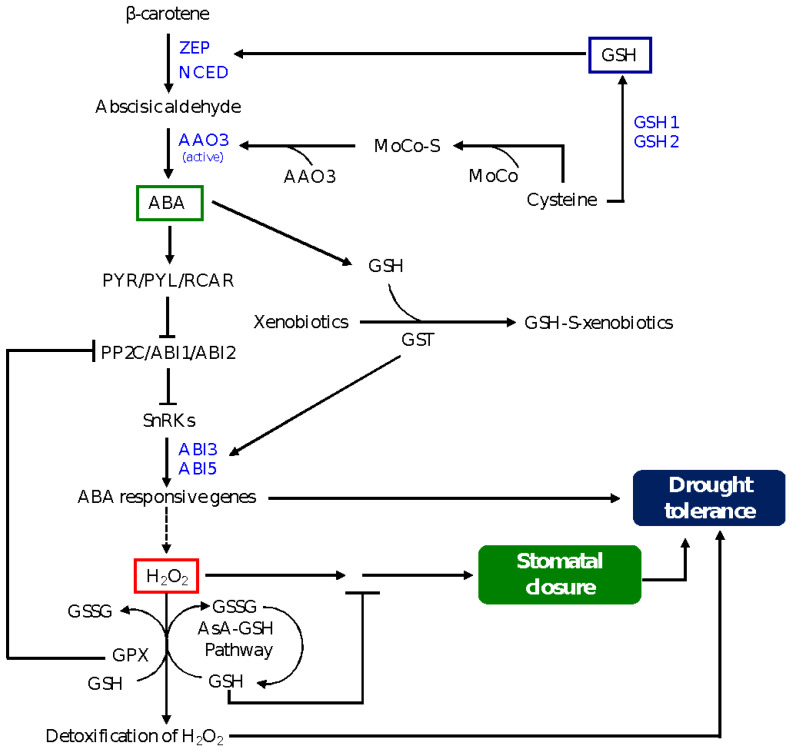
A model depicting GSH-mediated ABA signaling in stomatal closure and drought tolerance. Induction of GSH biosynthesis reduces the level of cysteine, which plays a role in activating the AAO3 enzyme and therefore ABA synthesis in the guard cells, leading to inhibition of ABA-induced stomatal closure. Whereas drought-induced GSH level enhances the expression levels of *ZEP* and *NCED*, resulting in an increase in ABA synthesis, GST detoxifies xenobiotic substances by conjugating them with GSH, leading to induction in the expression of genes encoding ABI3 and ABI5, transcription factors that directly activate the expression of ABA responsive genes and drought tolerance. GSH is involved in the regulation of the downstream signaling of ABA-induced H_2_O_2_ in the guard cells and thereby preventing stomatal closure. GSH is also involved in the detoxification of H_2_O_2_ through the AsA-GSH pathway. The role of GPX is as described in Figure 2, and it promotes ABA-induced stomatal closure and drought tolerance via inhibiting ABI2, which acts as a negative regulator of ABA signaling. ABA signaling components are described in Figure 2. ZEP, zeaxanthin epoxidase; NCED, 9-cis-epoxycarotenoid dioxygenase; AAO3 abscisic aldehyde oxidase3; MoCo, molybdenum cofactor; MoCo-S, sulfurylated MoCo; AsA, ascorbate.

A recent study has shown the significance of sulphur in ABA-mediated drought tolerance [71]. For example, maize roots accumulate high amounts of free sulphate, GSH and cysteine under drought conditions; accumulation of free sulphate promotes the synthesis and reduction of cysteine and induces the transcription of *GSH1*, which in turn improves the GSH redox state via increasing the total amount of GSH [108]. Previous studies have shown that loss of function mutation in *chloroplast sulfate transporter3;1*, which is involved in sulfate transport into chloroplasts, leads to a decrease in cysteine level [109]. Given that cysteine activates the transcription of the ABA biosynthetic gene *NCED3* and serves as a substrate for post-translational activation of the ABA biosynthesis enzyme AAO3 via MoCo sulfurylase ABA3, reduction in its level leads to inhibition of ABA synthesis [71]. These findings reflect the significance of modulation of GSH level in regulating ABA and drought stress signaling via post-translational regulatory mechanisms. A model shown in Figure 3 depicts the role of GSH in mediating ABA signaling in the regulation of stomatal closure and drought tolerance.

## 7. Conclusions and Future Prospects

Studies conducted to date have provided important insights into the role of GSH and ABA interplay at multiple levels of signaling cascades in the regulation of plant developmental events such as seed dormancy and germination, and their response to multiple environmental factors including drought. However, the molecular mechanisms underlying the role of GSH and ABA crosstalk in regulating these developmental and stress response events are still poorly understood. It is therefore necessary to identify the regulatory nodes that are central to ABA-GSH interaction in order to elucidate the hierarchy of the signal networks and understand the endogenous and exogenous cues that influence this hierarchy. Recent reports implicate GSH in the regulation of signaling pathways through modifying the respective protein components via *S*-glutathionylation and/or GSNO-mediated protein *S*-nitrosylation. Therefore, elucidating GSH-induced post-translational protein modifications of ABA and stress signaling components through biochemical, molecular and genetic studies will have paramount significance in enhancing our understanding of GSH-mediated regulation of ABA and stress signaling networks. Moreover, further studies are needed to identify the mechanisms underlying the synthesis, transport and degradation of GSNO and the roles of these processes in ABA-regulated plant developmental events and stress responses. GSH has also been implicated in the regulation of epigenetic mechanisms such as DNA and histone methylation that control transcriptional programs. Thus, exploring the role of GSH in epigenetic regulation of the expression of genes involved in the ABA and other signal transduction networks that control plant developmental processes such as seed dormancy, and plant response to stress factors such as drought, is essential to advance our knowledge on the topic.

## Data Availability

Not applicable.
